# Ustekinumab therapy for moderately to severely active pediatric Crohn’s disease: UNITI Jr study safety and efficacy results in patients weighing at least 40 kg

**DOI:** 10.1093/ecco-jcc/jjag011

**Published:** 2026-03-12

**Authors:** Elisabeth De Greef, Dan Turner, Jarosław Kierkuś, Bartosz Korczowski, Monika Meglicka, Stanley A Cohen, Jeffrey S Hyams, Anne M Griffiths, Joel R Rosh, Richard Strauss, Els Van Limbergen, Omoniyi J Adedokun, Lilianne Kim, Sheri Volger, for the UNITI Jr study Group, Pauline De Bruyne, Patrick Bontems, Ilse Hoffman, Francoise Smets, Sibylle Koletzo, Philip Bufler, Lena Woelfle, Tobias Wenzl, Ulrich Baumann, Denisa Pilic, Michael Melter, Orsolya Kadenczki, Csaba Bereczki, Antal Dezsofi, Ferenc Dicso, Erzsebet Szakos, Yigal Elenberg-Alter, Dror Shouval, Efrat Broide, Katsuhiro Arai, Takahiro Kudo, Takashi Ishige, Itaru Iwama, Tatsuki Mizuochi, Daiki Abukawa, Yuhki Koike, Hideki Kumagai, Sotaro Mushiake, Kinga Kowalska-Duplaga Piotr Landowski, Aelita Kamalova, Vladimir Kopeikin, Elena Sitnikova, Ekaterina Tsimbalova, Richard Russell, Sandhia Naik, Franco Torrente, Rafeeq Muhammed, Christine Spray, Michael Stephens, Tamara Feldman, Zarela Molle-Rios, Tiffany Linville, Thomas Sferra, Jeffrey Lewis, Otto Louis-Jacques, Marc Schaefer, Bankole Osuntokun, Bess Aramakis, Harold Couchoux, Kordian Von Cyga, Laurie Conklin, Bridget Godwin, Katarzyna Cieslak, Auguste Gaddah, Renping Zhang, Li Deng, Wouter van der Borght, Esau Moreno Carmache

**Affiliations:** KidZ Health Castle, Paediatric Gastroenterology, Hepatology, and Nutrition, UZ Brussel, Vrije Universiteit Brussel, Brussels, Belgium; The Juliet Keidan Institute of Pediatric Gastroenterology and Nutrition, The Eisenberg Research and Development Authority, Shaare Zedek Medical Center, The Hebrew University of Jerusalem, Jerusalem, Isreal; Department of Gastroenterology, Hepatology, Feeding Disorders and Paediatrics, The Children’s Memorial Health Institute, Warsaw, Poland; Department of Pediatrics and Pediatric Gastroenterology, Medical College, University of Rzeszów, Rzeszów, Poland; Department of Gastroenterology, Hepatology, Feeding Disorders and Paediatrics, The Children’s Memorial Health Institute, Warsaw, Poland; Children's Center for Digestive Health Care, Morehouse School of Medicine, Atlanta, GA, United States; Department of Gastroenterology, Connecticut Children’s Medical Center, Hartford, CT, United States; Inflammatory Bowel Disease Centre, The Hospital for Sick Children, University of Toronto, Toronto, Canada; Northwell Health, Division of Pediatric Gastroenterology, Liver Diseases and Nutrition, Cohen Children’s Medical Center, New Hyde Park, NY, United States; Quantitative Science Clinical Pharmacology, Johnson & Johnson, Spring House, PA, United States; Quantitative Science Clinical Pharmacology, Johnson & Johnson, Spring House, PA, United States; Quantitative Science Clinical Pharmacology, Johnson & Johnson, Spring House, PA, United States; Quantitative Science Clinical Pharmacology, Johnson & Johnson, Spring House, PA, United States; Quantitative Science Clinical Pharmacology, Johnson & Johnson, Spring House, PA, United States

**Keywords:** clinical trials, pediatrics, endoscopy

## Abstract

**Background:**

UNITI Jr is a phase 3 study evaluating the efficacy, safety, and pharmacokinetics of ustekinumab, an interleukin-12/-23 antagonist, in pediatric patients with moderately to severely active Crohn’s disease. Results from patients weighing at least 40 kg are reported.

**Methods:**

Patients at least 40 kg and aged less than 18 years with a Pediatric Crohn’s Disease Activity Index score of >30 received a single intravenous weight-tiered induction dose of ustekinumab. After 8 weeks, patients were randomized to subcutaneous ustekinumab 90 mg every 8 or 12 weeks. The primary endpoint was clinical remission at Week 8. Secondary endpoints included clinical response (Weeks 8, 52), endoscopic response (Weeks 16, 52), clinical remission, and corticosteroid-free remission (Week 52).

**Results:**

Of 48 patients, median age was 15.0 years (interquartile range: 14.0-16.0). At Week 8, 52.1% (25/48) achieved clinical remission and 93.8% (45/48) achieved clinical response. At Week 16, 29.8% (14/47) achieved endoscopic response. Clinical remission at Week 52 was achieved in 15/25 (60.0%; every 12 weeks) and 10/23 (43.5%; every 8 weeks). Three patients (6.3%) discontinued ustekinumab between Weeks 8 and 52. Ustekinumab was safe and well-tolerated; adverse event rates were similar between groups. Immunogenicity was low; trough median (mean) steady-state serum ustekinumab concentrations following 8 weeks of dosing were 1.38 (2.08) to 1.74 (2.32) μg/mL and were comparable to levels in adult patients: 2.83 (2.05) μg/mL.

**Conclusions:**

Ustekinumab induction and maintenance therapy was effective and safe through 52 weeks in pediatric patients weighing at least 40 kg with moderately to severely active Crohn’s disease.

**Trial Registration Numbers:**

NCT04673357

## Graphical abstract

**Graphical Abstract:**
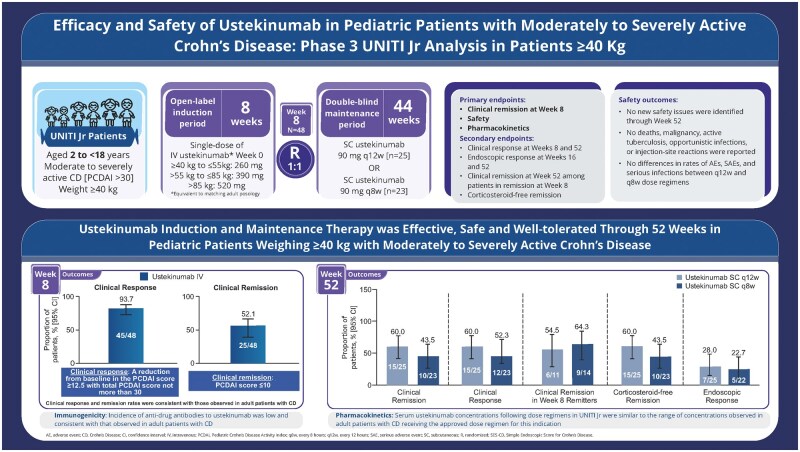

## 1. Introduction

The incidence of pediatric Crohn’s disease (CD) continues to increase with a global incidence of 2.5-11.4 per 100 000.^1^ Despite the availability of many biologic treatment options for adult CD, approved options for pediatric patients are limited.

Ustekinumab is a first-in-class fully human IgG1κ monoclonal antibody and the first biologic treatment to selectively inhibit the interleukin (IL)-12 and IL-23 cytokine pathways.^2^^,^^3^ Recently, the drug was approved in the European Union (EU) for the treatment of CD in pediatric patients <18 years weighing at least 40 kg.^4^ Additional health authority approvals are pending the full results of the UNITI Jr phase 3 study.

Previously, the randomized, double-blind, induction dose-ranging phase 1 UNISTAR study established the pharmacokinetics (PK), safety, and efficacy of intravenous (IV) ustekinumab induction followed by subcutaneous (SC) ustekinumab maintenance in pediatric patients with moderately to severely active CD.^5^ Data from the UNISTAR long-term extension study have confirmed the efficacy and PK of ustekinumab through 1 year and safety and immunogenicity through 4 years. Ustekinumab efficacy and safety in these pediatric patients with CD were generally comparable to results previously reported in adults.^6^

The pivotal, phase 3, UNITI Jr study is currently evaluating the efficacy, safety, and PK of ustekinumab IV induction and SC maintenance therapy in pediatric patients with moderately to severely active CD using adult-adjusted weight-based posology. These interim results supporting the EU filing for marketing authorization of ustekinumab in patients weighing at least 40 kg through Week 52 are reported here.

## 2. Methods

### 2.1. Study design

UNITI Jr (NCT04673357) is a multicenter, phase 3, open-label trial evaluating the efficacy, safety, and PK of induction therapy using a single IV ustekinumab dose followed by a randomized, double-blind phase of SC ustekinumab maintenance therapy in pediatric patients with moderately to severe active CD (Figure S1).

Data are presented from an interim analysis supporting the European Medicines Agency (EMA) submission of ustekinumab in pediatric CD for patients weighing ≥40 kg. All authors had access to the study data, and reviewed and approved the final manuscript. This study was conducted in accordance with the ethical principles that have their origin in the *Declaration of Helsinki* and that are consistent with Good Clinical Practices and applicable regulatory requirements.

### 2.2. Study population

Eligible pediatric patients weighed ≥40 kg and were <18 years old with moderately to severely active CD with a Pediatric Crohn’s Disease Activity Index (PCDAI) score of >30. All patients had an inadequate response and/or intolerance to biologic therapy (ie, tumor necrosis factor [TNF]α antagonist or vedolizumab) and/or conventional therapies (ie, IV or oral corticosteroids or immunomodulators azathioprine [AZA], 6-mercaptopurine [6-MP], and methotrexate [MTX]) or were corticosteroid-dependent. Patients were considered steroid-dependent if: (1) they failed a corticosteroid taper (ie, flare of disease) within 3 months of starting therapy, (2) if a relapse occurred within 3 months after discontinuing corticosteroids, or (3) they were unable to discontinue these agents without a loss of response (LOR) within 3 months. Patients had an ileocolonoscopy, performed within approximately 3 weeks prior to receiving induction dose, who showed evidence of active CD. Active CD was defined by the presence of ulceration and although a specific Simple Endoscopic Score for Crohn’s Disease (SES-CD) was not required, this was considered equivalent to an SES-CD score ≥3 during screening. Patients receiving enteral nutrition had to be on a stable regimen for at least 2 weeks prior to induction.

### 2.3. Study treatments

#### 2.3.1. Induction therapy

All patients received a single dose of IV ustekinumab at Week 0 using a weight-tiered induction dose equivalent to the approved adult dosing regimen (Figure S1): 260 mg IV for patients weighing ≥40 kg to ≤55 kg, 390 mg IV for those >55 kg to ≤85 kg and 520 mg IV for those >85 kg.

#### 2.3.2. Maintenance therapy

Patients completing the 8-week induction period (Week 8 visit) were randomized (1:1) into the 44-week maintenance period and received 90 mg SC ustekinumab either every 8 weeks (q8w) or every 12 weeks (q12w). Sham injections were utilized to maintain the blind. Randomization was stratified by response status at Week 8 (induction responder/induction nonresponder) and weight (<40 or ≥ 40 kg) (Figure S1).

Clinical responders who had an LOR and ustekinumab trough concentrations (≥1.4 μg/mL) were eligible to receive a dose adjustment (q12w to q8w) or sham dose adjustment (q8w remained on q8w) during the maintenance period from Week 16 through Week 52. Patients with confirmed LOR and relatively low steady-state trough ustekinumab concentrations (<1.4 µg/mL) during the maintenance period (Week 16 or Week 40) were eligible for the optional exposure optimization study (EOS) at an every 4-week (q4w) dosing regimen (EOS data not yet available for interim analysis).

### 2.4. Outcome measures

#### 2.4.1. Study endpoints

The global primary endpoints were clinical remission at Week 8, and safety and PK of ustekinumab. Secondary endpoints included clinical response at Week 8, and among patients who were clinical responders: clinical response at Week 16, endoscopic response at Week 16 and clinical remission, and endoscopic response at Week 52 along with clinical remission (among Week 8 remitters) at Week 52.

#### 2.4.2. Efficacy assessments

The PCDAI is a validated, clinician-reported, multi-item measure of disease activity designed for use in children with CD and was the primary endpoint for assessing disease activity response to ustekinumab.^7^^,^^8^ Clinical remission was defined as a PCDAI score ≤10 and clinical response was defined as a reduction from baseline in PCDAI score ≥12.5 points with a total PCDAI score not more than 30. Endoscopic response and improvement were assessed by ileocolonoscopy, which was performed at Screening, Week 16, and Week 52. Endoscopic response was measured using the SES-CD, defined by a reduction in SES-CD score ≥50% or SES-CD score ≤2, in patients with a baseline SES-CD score ≥3.^9^^,^^10^ An LOR was defined as an increase in PCDAI >12.5 above the reference PCDAI score and PCDAI score is >30 at two consecutive visits ≥7 days apart. Degree of inflammation was assessed using serum C-reactive protein (CRP) concentrations, fecal calprotectin, and fecal lactoferrin. Endpoint definitions are provided in Table S1. Growth during the study was assessed using age- and sex-specific *z*-scores for height, weight, and body mass index (BMI).

#### 2.4.3. Safety assessments

Types and incidence of adverse events (AEs), serious adverse events (SAEs), discontinuations, and infusion-related or injection-site reactions were documented. Malignancy, serious infections, and opportunistic infections were captured. Laboratory assessments included change from baseline in hematology and chemistry parameters according to National Cancer Institute Common Toxicity Criteria for AEs (NCI-CTCAE v5.0).^11^

#### 2.4.4. Pharmacokinetics and immunogenicity

Serum ustekinumab concentrations were measured over time. Anti-drug antibodies to ustekinumab (positive or negative) were evaluated using serum samples collected from all patients and titers of confirmed positive samples were reported. Additionally, the ability of the antibodies to neutralize the activity of ustekinumab was also measured.

### 2.5. Statistical analysis

This interim analysis supporting the EMA submission was based on at least 36 randomized patients weighing ≥40 kg completing both the induction and maintenance periods of the study or if they terminated the maintenance phase prior to Week 52. Fisher Information Matrix (FIM) methods were used to optimize the number of patients and PK samples to support the earlier regulatory submission and approval in the EU in pediatric patients. Descriptive statistics including number of observations, mean, standard deviation (SD), median, and interquartile range (IQR, minimum and maximum) were used to summarize continuous variables. Numbers and percentages were used to summarize categorical values. Unless otherwise specified, the Wilson score method was used to construct the 95% confidence interval (CI) for binary endpoints where a proportion and the respective 95% CI were reported. Patients ≥40 kg at both Weeks 0 and 8 who received at least one dose of ustekinumab during the maintenance period and completed the Week 52 visit (or terminated the study prior to Week 52) were included in the intent-to-treat analysis. Patients included in the PK analysis population had PK samples collected at Weeks 0, 8, and 16.

#### 2.5.1. Missing efficacy data

The total PCDAI score could only be calculated when three or more of the five PCDAI disease activity components were available at the specified visit. Missing values were carried forward from the screening visit in accordance with standard practice for phase 3 pediatric clinical trials.^12^ If fewer than three components were available, the PCDAI could not be calculated and was considered missing, and the patients were categorized to experience treatment failure (nonresponder). If three or more of the five component scores were available, but individual item data were missing from the remaining component, the points assigned to the missing item were determined by carrying forward the item value from the previous visit (Table S2).

## 3. Results

### 3.1. Patients

**Figure 1.**
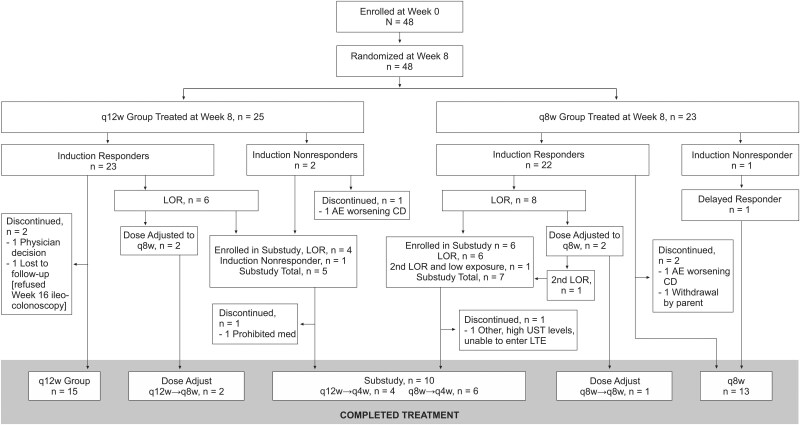
Patient disposition. AE, adverse event; CD, Crohn’s disease; LOR, loss of response; LTE, long-term extension; q4w, every 4 weeks; q8w, every 8 weeks; q12w, every 12 weeks; UST, ustekinumab.

Between April 11, 2021 and January 22, 2024, a total of 48 patients weighing ≥40 kg were enrolled and given weight-tiered IV induction followed by randomization to SC ustekinumab 90 mg q12w (*n* = 25) or 90 mg q8w (*n* = 23). Patients were recruited from 16 centers across seven countries including Poland (60.4%), Belgium (20.8%), Hungary (8.3%), USA (4.2%), Germany (2.1%), UK (2.1%), and Japan (2.1%). Five (10.4%) discontinued the study during the maintenance period, including three (12%) and two (8.7%) in the q12w and q8w groups, respectively (Figure 1). Reasons for discontinuation were AE of worsening of CD (*n* = 2), lost to follow-up, physician decision, and withdrawal by parent or guardian (*n* = 1 each).

Baseline clinical disease characteristics were generally well balanced across treatment groups and are representative of a population with moderately to severely active CD (Table 1). The median age of patients was 15.0 years (IQR: 14.0, 16.0), median weight 53.8 kg, and median BMI *z*-score was −0.16. However, some minor differences were observed between groups in that there was a greater proportion of males (69.6%) than females (30.4%) in the q8w group and the median BMI *z*-score was 0.17 (−0.7; 2.6) for females and −0.26 (−1.7; 4.1) for males.

**Table 1.** Baseline demographics and disease characteristics.

Characteristic^a^	Ustekinumab IV to SC q12w (*n* = 25)	Ustekinumab IV to SC q8w (*n* = 23)	Total (*N* = 48)
**Age, mean (SD), years**	14.7 (1.52)	14.7 (1.76)	14.7 (1.62)
**Age, median (IQR), years**	14.0 (14.0-16.0)	15.0 (14.0-16.0)	15.0 (14.0-16.0)
**Age range, years**	11-17	10-17	10-17
**Sex, *n* (%)**			
** Female**	14 (56.0)	7 (30.4)	21 (43.8)
** Male**	11 (44.0)	16 (69.6)	27 (56.3)
**Race, *n* (%)**			
** White**	22 (88.0)	22 (95.7)	44 (91.7)
** Asian**	2 (8.0)	1 (4.3)	3 (6.3)
** Black**	1 (4.0)	0	1 (2.1)
**Weight, kg, median (range)**	47.8 (41-83)	55.4 (42-85)	53.8 (41-85)
**Height *z*-score,^b^ median (range)**	0.67 (−1.4 to 1.5)	0.47 (−1.3 to 2.3)	0.62 (−1.4 to 2.3)
** Female**	0.60 (−1.4 to 1.3)	0.84 (−1.2 to 2.0)	0.68 (−1.4 to 2.0)
** Male**	0.68 (−0.7 to 1.5)	0.37 (−1.3 to 2.3)	0.38 (−1.3 to 2.3)
**BMI, kg/m^2^, median (range)**	18.7 (14-29)	19.7 (16-30)	19.5 (14-30)
**BMI *z*-score,^b^ median (range)**	−0.26 (−1.7 to 2.6)	0.08 (−1.3 to 4.1)	−0.16 (−1.7 to 4.1)
** Female**	−0.31 (−0.7 to 2.6)	0.18 (−0.3 to 0.5)	0.17 (−0.7 to 2.6)
** Male**	−0.26 (−1.7 to 1.0)	−0.31 (−1.3 to 4.1)	−0.26 (−1.7 to 4.1)
**Age at diagnosis, years, mean (SD)**	12.1 (3.11)	12.2 (2.50)	12.1 (2.80)
**Disease duration, years, mean (SD)**	2.7 (2.65)	2.6 (1.91)	2.6 (2.30)
**Involved GI areas, *n* (%)**			
** Ileum only**	2 (8.0)	4 (17.4)	6 (12.5)
** Colon only**	8 (32.0)	6 (26.1)	14 (29.2)
** Ileum and colon**	10 (40.0)	9 (39.1)	19 (39.6)
** Proximal small intestine, stomach and/or esophagus**	14 (56.0)	8 (34.8)	22 (45.8)
** Perianal**	6 (24.0)	7 (30.4)	13 (27.1)
**PCDAI score, mean (SD)**	42.0 (9.04)	41.2 (6.02)	41.6 (7.68)
**PCDAI score, median (range)**	40.0 (25.0-62.5)	40.0 (32.5-55.0)	40.0 (25.0-62.5)
**SES-CD score, mean (SD)**	12.5 (6.33)	12.1 (7.43)	12.3 (6.81)
**SES-CD score, median (range)**	11.0 (3-26)	12.0 (0-34)	11.5 (0-34)
**IMPACT III, mean (SD)**	102.6 (25.05)^c^	98.6 (22.57)^d^	100.7 (23.72)^e^
**CRP, mg/L, median (IQR)**	10.7 (5.60-41.20)	13.1 (2.20-41.90)	11.4 (4.40-41.30)
**Fecal calprotectin, mg/kg, median (IQR)**	1971.0 (1739.0-3725.0)	1874.5 (966.0-3780.0)^c^	1971.0 (993.0-3780.0)^f^

a Note: characteristics were descriptively compared; no formal statistical analysis was conducted between treatment groups.

b Age- and sex-specific.

c Data available for only 22 patients.

d Data available for only 19 patients.

e Total of 41 patients.

f Total of 47 patients.

Abbreviations: BMI, body mass index; CD, Crohn’s disease; CDAI, Crohn’s Disease Activity Index; CRP, C-reactive protein; GI, gastrointestinal; IQR, interquartile range; PCDAI, Pediatric CD Activity Index; sPCDAI, Short Pediatric CD Activity Index; SES-CD, Simple Endoscopic Score for Crohn’s Disease.

Overall, the mean duration of CD was 2.6 years and mean PCDAI score was 41.6. The mean SES-CD score was 12.3. The median fecal calprotectin level was 1971.0 mg/kg and median CRP concentration was 11.4 mg/L.

At baseline, 72.9% of patients were receiving CD-related medications. The types of CD medications being used at baseline by patients were generally well-balanced across treatment groups, except the greater percentage of patients with corticosteroid use (Table 2). Twenty-seven patients (56.3%) were biologic-naïve with no prior history of use and 23 (47.9%) patients had prior use of an immunomodulator (6-MP, AZA, or MTX) and experienced an inadequate response or intolerance.

**Table 2.** CD-related medications and therapies.

Treatment, *n* (%)	Ustekinumab IV to SC q12w (*n* = 25)	Ustekinumab IV to SC q8w (*n* = 23)	Total (*N* = 48)
**Prior history**			
** History of inadequate response or intolerance to biologics**	9 (36.0)	10 (43.5)	19 (39.6)
** No history of inadequate response or intolerance to prior biologics**	16 (64.0)	13 (56.5)	29 (60.4)
**Biologic-naïve**	15 (60.0)	12 (52.2)	27 (56.3)
** Biologic-experienced, but no documented reason for stopping therapy**	1 (4.0)	1 (4.3)	2 (4.2)
**History of inadequate response or intolerance to prior immunomodulators^a^**	13 (52.0)	10 (43.5)	23 (47.9)
** Prior nutritional therapy^b^**	16 (64.0)	15 (65.2)	31 (64.6)
** EN therapy as a primary therapy**	11 (44.0)	9 (39.1)	20 (41.7)
** Partial EN as a primary therapy^c^**	10 (40.0)	7 (30.4)	17 (35.4)
**Baseline use**			
** CD-related concomitant medications**	18 (72.0)	17 (73.9)	35 (72.9)
** Corticosteroids^d^**	6 (24.0)	8 (34.8)	14 (29.2)
** Immunomodulators^a^**	9 (36.0)	9 (39.1)	18 (37.5)
** 5-ASA**	11 (44.0)	10 (43.5)	21 (43.8)

a Immunomodulators included AZA, 6-MP, and MTX.

b Patients could have had more than one prior nutritional therapy.

c Partial EN is defined as <80% of total nutritional requirements given as enteral tube feeding in patients whose oral intake of food and fluids is inadequate to meet their nutritional needs.

d Patients were on a stable dose of corticosteroids for at least 2 weeks prior to receiving induction therapy (no IV or rectal corticosteroids were permitted).

Abbreviations: 5-ASA, 5-aminosalicylates; 6-MP, 6-mercaptopurine; AZA, azathioprine; CD, Crohn’s disease; EN, enteral nutrition; IV, intravenous; MTX, methotrexate; q8w, every 8 weeks; q12w, every 12 weeks; SC, subcutaneous.

A total of 14 (29.2%) of 48 patients had a confirmed LOR (defined by a normal ustekinumab trough level ≥1.4 µg/mL) and four (8.3%) had a dose adjustment or sham adjustment from q12w (*n* = 2) and q8w (*n* = 2) to q8w dosing regimen during the maintenance period.

### 3.2. Efficacy

#### 3.2.1. Efficacy data

All patients who completed the Week 8 and Week 52 visit had PCDAI scores recorded and computed for these visits. Full PCDAI clinical data were available for all patients. Missing laboratory data at Week 8 (eg, hematocrit, erythrocyte sedimentation rate, or albumin) were carried forward from the screening visit in nine patients (18.8%) and used to compute the PCDAI subcomponent laboratory score.

#### 3.2.2. Induction endpoints

**Figure 2.**
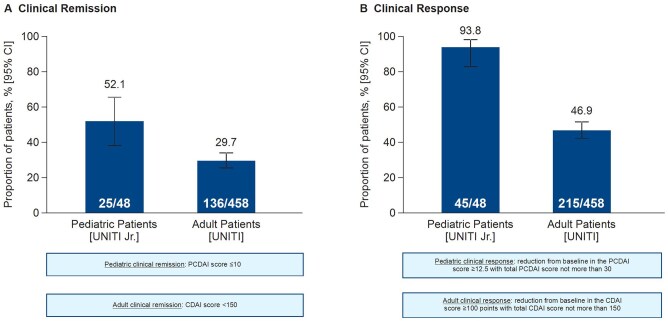
Clinical remission (primary endpoint) and clinical response at Week 8. Note: outcomes in adult patients were obtained from the UNITI trial.^13^ CDAI, Crohn’s Disease Activity Index; CI, confidence interval; PCDAI, Pediatric Crohn’s Disease Activity Index.

**Figure 3.**
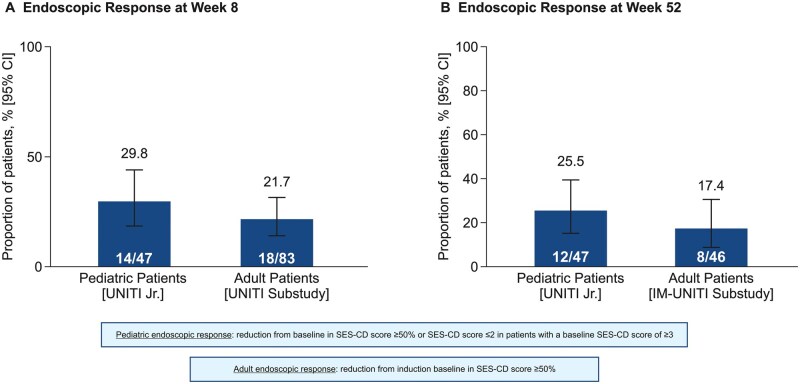
Comparison of endoscopic response in pediatric and adult CD patients. Note: outcomes in adult patients were obtained from a substudy of the UNITI/IM-UNITI trials.^19^ CI, confidence interval; SES-CD, Simple Endoscopic Score for Crohn’s Disease.

The primary endpoint, clinical remission at Week 8, was achieved by 25 of 48 patients (52.1%; 95% CI: 38.3%, 65.5%) (Figure 2). All sensitivity and supplementary analyses for the primary outcome had identical results as the main analysis due to lack of intercurrent events and completeness of the PCDAI data. At Week 8, 93.8% of patients (95% CI: 83.2%, 97.9%) achieved clinical response (Figure 2). An endoscopic response, which was evaluated at Week 16 (8 weeks after completion of induction), was achieved in 29.8% (95% CI: 18.7%, 44.0%) (Figure 3). At Week 16, 43 (89.6%) patients (95% CI: 77.8%, 95.5%) achieved clinical response.

#### 3.2.3. Maintenance endpoints

Overall, at the end of the maintenance period (Week 52), 52.1% (95% CI: 38.3%, 65.5%) achieved clinical remission and 56.3% (95% CI: 42.3%, 69.3%) achieved clinical response. In addition, 52.1% (95% CI: 38.3%, 65.5%) also achieved corticosteroid-free clinical remission. Among the 14 patients who were receiving corticosteroids at baseline (Week 0), eight (57.1%) achieved corticosteroid-free clinical remission after 44 weeks of maintenance therapy.

**Figure 4.**
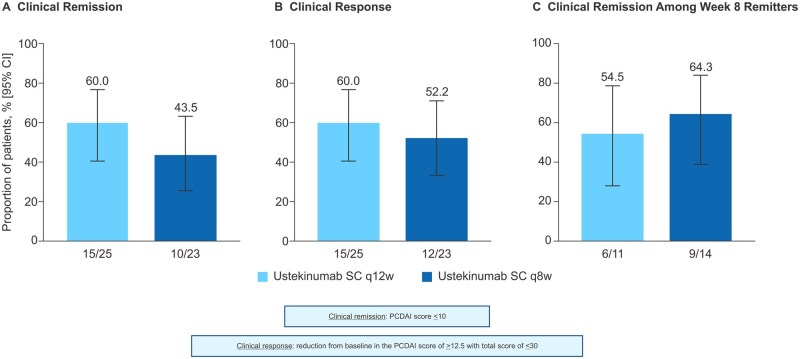
Clinical outcomes at Week 52 by treatment group. CI, confidence interval; PCDAI, Pediatric Crohn’s Disease Activity Index; q8w, every 8 weeks; q12w, every 12 weeks; SC, subcutaneous.

Regardless of treatment group (ustekinumab 90 mg SC q12w or 90 mg SC q8w), the proportion of patients achieving clinical remission and clinical response after 44 weeks of maintenance therapy was similar (Figure 4). In addition, clinical remission at Week 52 among Week 8 remitters was achieved in six (54.5%) of 11 patients (95% CI: 28%, 78.7%) in the q12w treatment group and nine (64.3%) of 14 patients (95% CI: 38.8%, 83.7%) in the q8w treatment group (Figure 4). At Week 52, the proportion of patients in clinical remission among patients in clinical remission at Week 8 (60.0%, [15/25]; 95% CI: 40.7%, 76.6%), was similar to that of the adult patients on ustekinumab SC 90 mg (66.7% [52/78]; 95% CI: 55.6%, 76.1%) but higher than that of adult patients on placebo (45.6% [36/79]; 95% CI: 35.0%, 56.5%) (data not shown).^13^ The exposure optimization substudy was still ongoing at the time of database lock.

##### Subgroup

The proportions of patients in clinical remission at Week 8 were 62.1% in those without a history of failure to treatment with biologics (failed conventional therapy) at baseline and 36.8% in those with a history of biologic failures at baseline. At Week 52, the proportions of pediatric patients who achieved clinical remission and clinical response were higher in the nonbiologic failure subgroup with similar proportions of patients (19; 65.5% for each) achieving clinical remission, clinical response, and corticosteroid-free clinical remission compared with six (31.6%), eight (42.1%), and six (31.6%), respectively, in the biologic failure group.

##### Biomarkers

The mean (SD) decrease from baseline in CRP concentration at Week 8 was 13.89 mg/L (25.825) and was maintained through Week 52. Overall, the mean (SD) decreases in CRP concentrations were 11.46 mg/L (27.969) in the q12w group and 10.85 (19.832) in the q8w group. In a post-hoc analysis of 34 patients with elevated CRP levels (>5 mg/L) at baseline, 7/30 (23.3%) had a normalized level at Week 8. Five of 14 patients (35.7%) in the q12w treatment group and 5/15 patients (33.3%) in the q8w group had normalized CRP levels at Week 52, for a combined CRP normalization rate of 10 of 29 (34.5%).

At Week 8, of 41 patients, nine (22.0%) had normalization of fecal calprotectin levels (≤250 mg/kg). At Week 52, fecal calprotectin levels normalized in 6/41 (14.6%) patients with 1/22 (4.5%) and 5/19 (26.3%) in the q12w and q8w groups, respectively. Clinical biomarker responses (for the combined group) were 47.9% at Week 16 and 39.6% at Week 52 (after 44 weeks of maintenance therapy).

### 3.3. Safety

#### 3.3.1. Induction period

Patients were followed for an average of 8.3 weeks. During the induction period, 31 patients (64.6%) reported one or more AEs. One (2.1%) patient with a prior history of nephrolithiasis experienced an SAE of nephrolithiasis, which was considered unrelated to treatment and resulted in hospitalization through Week 8. Three (6.3%) AEs were considered treatment-related (nausea, headache, and psoriasis).

#### 3.3.2. Maintenance period

The average duration of follow-up was 37.4 weeks for the maintenance period. Between the q12w and q8w treatment groups, the rates of AEs, SAEs, and AEs leading to discontinuation (<5% in both groups) were similar during the SC maintenance period (Table 3). AEs were reported by 92.0% and 91.3% of patients in the q12w and q8w groups, respectively. SAEs were reported in four (16%) patients receiving q12w and in four (17.4%) receiving q8w. Serious AEs included CD, bloody diarrhea, excision of a perianal fistula, gastritis due to *Aeromonus* infection, suicide attempt (in a patient with a history of untreated depression), elevated liver enzymes (probably due to muscle damage from vigorous exercise), broken right clavicle, and syncope. Infections were reported at a similar rate between groups (68% for q12w, 69.6% for q8w), none of which were considered serious. No injection site reactions were reported. Two isolated grade 3 laboratory abnormalities were reported (lymphopenia and increased aspartate aminotransferase). No patients experienced a grade 4 laboratory abnormality.

**Table 3.** Adverse events through week 52.

	Ustekinumab IV to SC q12w (*n* = 25)	Ustekinumab IV to SC q8w (*n* = 23)	Combined (*N* = 48)
**Average duration of follow-up (weeks)**	35.5	39.4	37.4
**Average number of administrations**	6.3	6.8	6.5
**Patients with 1 or more, *n* (%)**			
** AEs**	23 (92.0)	21 (91.3)	44 (91.7)
** SAEs**	4 (16.0)	4 (17.4)	8 (16.7)
** Death**	0	0	0
** AEs leading to discontinuation**	1 (4.0)	1 (4.3)	2 (4.2)
** AEs of severe intensity**	2 (8.0)	0	2 (4.2)
** Infections**	17 (68.0)	16 (69.6)	33 (68.8)
** Serious infections**	0	0	0
** Infections requiring oral and/or parenteral antimicrobial treatment**	2 (8.0)	7 (30.4)	9 (18.8)
** Malignancy**	0	0	0
** Active tuberculosis**	0	0	0
** Opportunistic infections**	0	0	0
** Injection site reactions^a^**	0	0	0

a Includes any reaction at SC intervention injection site recorded as an injection-site reaction by the investigator on the electronic case report form.

Abbreviations: AE, adverse event; q8w, every 8 weeks; q12w, every 12 weeks; SAE, serious adverse event.

### 3.4. Pharmacokinetics and immunogenicity

#### 3.4.1. Induction period

After a single dose of IV ustekinumab (approximately 6 mg/kg), the median (IQR) peak serum ustekinumab concentration, which was observed approximately 1 h after the end of the infusion, was 91.8 μg/mL (84.1; 111.8). At Week 8 (main efficacy assessment), the median (IQR) serum ustekinumab concentration decreased to 3.38 μg/mL (1.74; 6.2).

#### 3.4.2. Maintenance period

Prior to administration of the first maintenance dose of ustekinumab, median (IQR) trough serum ustekinumab concentrations were 3.13 μg/mL (1.52; 5.32) and 3.38 μg/mL (1.95; 7.97) in patients receiving SC ustekinumab 90 mg q12w and q8w, respectively. In both maintenance dosing groups, ustekinumab serum concentrations were sustained through Week 52. Steady-state concentrations of ustekinumab were reached at approximately 16 and 20 weeks after IV induction in patients receiving SC ustekinumab 90 mg maintenance dosing of q8w or q12w, respectively. Irrespective of administration schedule (SC q8w or q12w), there was no apparent accumulation in ustekinumab concentration over time.

During the maintenance period, the median trough serum ustekinumab concentrations were consistent and ranged from 0.41 to 0.67 μg/mL (mean 0.58-0.65 μg/mL) in the 90 mg SC q12w group and from 1.38 to 1.74 μg/mL (mean 2.08-2.57 μg/mL) in the 90 mg SC q8w dosing group. Steady-state trough ustekinumab concentrations in patients who received q8w dosing were approximately 3-fold the concentrations of those who received q12w dosing. At the end of the maintenance period where key efficacy endpoints were evaluated, the median (IQR) serum ustekinumab concentrations were 1.76 μg/mL (1.44; 2.44) in the q12w group and 6.11 μg/mL (4.21; 7.59) in the q8w group.

Five patients in the q12w group and seven in the q8w dose regimen who experienced LOR with low ustekinumab levels (<1.4 µg/mL) entered the EOS, where the ustekinumab dose regimen was adjusted to q4w. The q4w dosing regimen led to a higher steady-state exposure compared with q12w and q8w dosing regimens. However, ustekinumab exposures in these pediatric patients who received q4w dosing were within the range of the exposures observed in adult participants receiving the approved q8w dosing.

#### 3.4.3. Immunogenicity

There were 46 patients who received ustekinumab during induction and maintenance therapy who had appropriate samples drawn. Through Week 52, only one (2.2%) was positive for anti-drug antibodies to ustekinumab (peak titer of 1:200) and was also positive for neutralizing antibodies.

## 4. Discussion

Results from this interim analysis of the phase 3 UNITI Jr trial provide evidence that ustekinumab is a safe and effective therapy in pediatric patients weighing ≥40 kg with moderately to severely active CD whose disease had previously experienced treatment failure or were intolerant to conventional and/or biologic therapy. As a result of this analysis, in April 2025, ustekinumab was approved in the EU for the treatment of CD in pediatric patients weighing 40 kg or more^4^; however, approval for younger children (weighing less than 40 kg) and approval in the USA and the rest of the world for children 2 to less than 18 years, of any weight, is pending. As anti-TNF therapies remain the only approved biologics for children, there remains an urgent unmet need for additional safe, effective, and convenient treatment options for all pediatric patients with CD, but especially for patients whose CD has failed treatment with existing therapies.

Clinical data on the use of ustekinumab in pediatric patients with CD are limited, primarily comprising single-center or observational studies without standardized treatment protocols.^14–17^ The only other prior registrational trials examining the use of ustekinumab in this population are the UNISTAR 16-week PK study and its long-term extension study.^5^^,^^6^

The UNISTAR pediatric study lacked a control group; however, given that CD in children is similar to that in adults in clinical presentation, pathophysiology, and expected response to ustekinumab, there is strong evidence to support the extrapolation of efficacy and safety from adults to pediatric patients weighing at least 40 kg and under <18 years. Efficacy and safety of ustekinumab in the treatment of adults with moderate to severely active CD has been established in the UNITI-1 and UNITI-2 single IV dose (approximately 6 mg/kg) induction studies and IM-UNITI SC maintenance therapy study (90 mg every 12 or 8 weeks) with long-term extension data out to 5 years.^13^^,^^18^ The ustekinumab dosing regimens used in UNITI Jr paralleled the doses according to the adult label.^2^^,^^3^ Ustekinumab induction therapy given to pediatric patients resulted in a numerically higher rate of clinical remission and clinical response compared with adult patients who participated in the Phase 3 UNITI-1 and UNITI-2 induction studies. However, the definitions of clinical remission (CDAI score <150) and clinical response (decrease from baseline CDAI score ≥100 points or a total CDAI score <150) in these adult studies varied compared with those used in UNITI Jr, which used the PCDAI tool to measure clinical remission and response (Figure 2).^13^

Clinical remission at Week 52 in pediatric patients on maintenance ustekinumab therapy was similar to that observed in the IM-UNITI adult maintenance study (data not shown).^13^ However, despite the small sample size in the pediatric study, both q12w and q8w dosing regimens were found to be beneficial, with adult maintenance outcomes for q8w (68/128; 53.1%) numerically higher compared with the q12w regimen (63/129; 48.8%), though the CIs overlapped. In spite of the comparable efficacy demonstrated in pediatric patients compared to adults for both q12w and q8w dosing regimens, there were still some patients (12/48; 25%) who had low exposures and either failed to initially respond or lost response during maintenance therapy with either regimen. The high proportion of pediatric patients who were eligible for the EOS and dose adjustment to q4w due to low exposure suggests the importance of individualizing treatment approaches by optimizing dosing in patients who have not responded or have lost response to treatment.

The proportion of pediatric patients who were in endoscopic response at Week 16 was 29.8% and was numerically greater than the proportion of adult patients in the non-randomized endoscopic substudy (21.7%; Figure 3).^19^ Furthermore, in pediatric patients, a clinically meaningful (61.7%) endoscopic improvement from baseline at 16 weeks was observed and persisted through 44 weeks of maintenance therapy. Of note, the UNITI Jr trial did not specify a minimum baseline SES-CD score as an inclusion criterion. Eligibility required only the presence of ulceration on ileocolonoscopy, which typically corresponds to an SES-CD score of approximately 3. The omission of a quantitative SES-CD threshold was done to align with the adult ustekinumab program, which did not have an SES-CD inclusion criterion. Such alignment is critical under the extrapolation concept. This design choice permitted enrollment of pediatric patients with mild disease activity, and thereby might lead to increased heterogeneity of the study population. Although analyses were restricted to those with a baseline SES-CD of ≥3, this cut-off may still include patients with mild disease and thus limits the generalizability of these findings to patients with moderate to severe disease. Consequently, the reported endoscopic response rates should be interpreted with caution.

The efficacy results with maintenance therapy from UNITI Jr are reported in all randomized patients (ie, responders and nonresponders), while the adult results are reported for responders only.^13^ Nevertheless, overall, clinical remission, clinical response, and changes in inflammatory biomarkers (CRP and fecal calprotectin) were consistently similar between pediatric and adult CD studies of ustekinumab after 44 weeks of maintenance therapy (Table S3).^13^ In patients with CD with moderate to severe symptoms, the American Gastroenterological Association (AGA) recommends a level of >5 mg/L for CRP (or fecal calprotectin >150 µg/g) to “rule in” active inflammation.^20^ They suggest using these biomarker thresholds to inform treatment decisions in an attempt, especially relevant in children, to avoid the more invasive endoscopic assessment of disease activity. The STRIDE II guidelines also recommend a level of >5 mg/L for CRP.^21^ Similar to the adult UNITI trial, the UNITI Jr study employed a more stringent CRP normalization criterion, defining normalization as CRP ≤ 3 mg/L, and incorporated intercurrent event (ICE) criteria in the analysis, thereby facilitating the reporting of these results in a consistent manner across pediatric and adult studies. Without ICE criteria applied to CRP, there were 8/31 (25.8%) who achieved a CRP level <3 mg/L and 10/29 (34.5%) who were <5 mg/L in UNITI Jr at Week 52 (Table S3). Using the STRIDE II recommended CRP threshold of <5 mg/L rather than the study mandated threshold of 3 mg/L demonstrated a somewhat smaller denominator but a larger proportion of patients who achieved normalization of CRP. Although a direct comparison cannot be made between studies, the efficacy results observed in UNITI Jr are similar to those reported in the open-label REACH trial of infliximab in pediatric patients with moderately to severely active CD who were biologically naïve.^22^

Prolonged corticosteroid administration in pediatric patients can result in known adverse consequences, especially with regard to negative effects on linear growth, and therefore reducing corticosteroid dosing is an important treatment goal in this patient population. Notably, the proportion of pediatric patients in corticosteroid-free clinical remission at Week 52 was 52.1% and was numerically higher than the 43.2% reported in the adult CD study.^13^ Results from UNISTAR provide additional support for the safety and efficacy of ustekinumab in pediatric patients, with 38.2% of patients achieving corticosteroid-free clinical remission at Week 48.^6^ Similarly, in the largest real-world use of ustekinumab in pediatric patients with CD to date, the STEP-CD retrospective analysis from the IBD Porto group in those aged 2-18 years observed a 40.5% corticosteroid-free clinical remission rate at Week 52.^17^ Furthermore, real-world data from two large centers on the use of ustekinumab in pediatric CD (active CD with sPCDAI ≥ 15, CRP ≥ 1.5 mg/dL, and/or fecal calprotectin ≥250 µg/g at the time of ustekinumab initiation) demonstrated that a high proportion of patients received clinical benefit, with 80% of patients in steroid-free clinical remission at 1 year (primary objective) with rare reports of AEs.^14^

In general, ustekinumab IV induction and 44 weeks of SC maintenance therapy was safe and well-tolerated by pediatric participants weighing ≥40 kg with moderately to severely active CD. This included the weight-based IV induction dose and 90 mg SC at both q12w and q8w dosing regimens for ustekinumab. No new safety signals were observed during the UNITI Jr trial. No deaths, serious infections, or opportunistic infections occurred during the induction or maintenance periods. Importantly, no notable differences in the rates of AEs, SAEs, and serious infections between the q12w and q8w dose regimens were observed. The safety profile of ustekinumab observed in this >40-kg subpopulation from the UNITI Jr trial was comparable to that established for the approved dose for adult CD and pediatric psoriasis indications.^2^^,^^3^

Following the dosing regimens used in this study, serum ustekinumab concentrations were within the range of concentrations observed in adult patients with moderately to severely active CD receiving the approved dosing regimen for this indication.^13^ The incidence of anti-drug antibodies to ustekinumab observed was low during both induction and maintenance treatment and was consistent with the immunogenicity profile of ustekinumab in adult patients with CD.^13^

Other limitations of this study include a lack of a placebo control group along with a relatively small sample size and too few patients to definitively show the benefits of dose escalation (q12w to q8w, or q8w to q4w). Also, while PCDAI scores were available for this analysis, nine patients had missing laboratory PCDAI subscore values at the assessment visit. In accordance with the a priori analysis plan, missing laboratory parameters were imputed using the last observation carried forward (LOCF) method. This approach is standard in pediatric CD trials, where laboratory values from the previous visit are carried forward and used to compute the laboratory subscore. It is considered a conservative method as it assumes no improvement in the laboratory values. Thus, it is unlikely that LOCF methods contribute significantly to variability in response given that laboratory data represent only a small component of the total PCDAI score. Finally, because of differences in PCDAI and CDAI for use in assessing clinical endpoints and data collection requirements used between studies, including differences in required versus optional endoscopy performance, direct comparisons between adult and pediatric ustekinumab studies should be interpreted with some caution.

Based on this trial, ustekinumab has demonstrated efficacy in both the biologic-naïve and biologic-failure population, allowing earlier initiation of therapy (vs use only as a salvage therapy). Furthermore, ustekinumab has a well-known and favorable safety profile. In conclusion, the safety and efficacy data from this interim analysis of UNITI Jr demonstrate a positive benefit versus risk profile that supported EMA marketing authorization for induction with IV ustekinumab and SC maintenance dosing in pediatric patients weighing ≥40 kg with moderately to severely active CD.

## Conference presentations

The results reported in this publication were presented at DDW 2025 (Digestive Disease Week^®^), May 3-5, 2025, San Diego, CA. The following abstract is published in a supplement of *GIA: Gastroenterology Endoscopy*, pp. S1-S756 (May 2025).

- De Greef E, Strauss R, Van Limbergen E, et al. Ustekinumab open-label induction and randomized blinded maintenance therapy in pediatric participants with moderately to severely active Crohn’s disease: The phase 3 UNITI Jr study. Oral Presentation #4243327.
  These results were also presented as an encore at ESPGHAN (European Society for Paediatric Gastroenterology, Hepatology and Nutrition) 57th Annual Meeting, May 14-17, 2025, Helsinki, Finland. The following abstract is published in an abstract book in the *Journal of Pediatric Gastroenterology and Nutrition* (JPGN).
- De Greef E, Strauss R, Van Limbergen E, et al. Ustekinumab open-label induction and randomized blinded maintenance therapy in paediatric participants with moderately to severely active Crohn’s disease: The phase 3 UNITI Jr Study. Poster #G‐OP044.

## Supplementary Material

jjag011_Supplementary_Data

## Data Availability

The data sharing policy of Johnson & Johnson is available at https://innovativemedicine.jnj.com/our-innovation/clinical-trials/transparency. As noted on this site, requests for access to the study data can be submitted through Yale Open Data Access (YODA). Project site at http://yoda.yale.edu.
