# Supplementary material for: Ustekinumab therapy for moderately to severely active pediatric Crohn’s disease: UNITI Jr study safety and efficacy results in patients weighing at least 40 kg
Source: J Crohns Colitis. 2026 Mar 12;20(3):jjag011. doi: 10.1093/ecco-jcc/jjag011 (PMC13017028; doi:10.1093/ecco-jcc/jjag011)
Supplement: jjag011_Supplementary_Data [file jjag011_supplementary_data.zip › Supplement_Data.zip!/Supplementary Info/UNITI Jr 40 kg Supplementary Figure S1 2.23.26.docx]

**Supplementary Figure S1. Study design**

IV, intravenous; kg, kilogram; q4w, every 4 weeks; q8w, every 8 weeks; q12w, every 12 weeks; SC, subcutaneous; µg/mL, microgram per milliliter.
