# Supplementary material for: Ustekinumab therapy for moderately to severely active pediatric Crohn’s disease: UNITI Jr study safety and efficacy results in patients weighing at least 40 kg
Source: J Crohns Colitis. 2026 Mar 12;20(3):jjag011. doi: 10.1093/ecco-jcc/jjag011 (PMC13017028; doi:10.1093/ecco-jcc/jjag011)
Supplement: jjag011_Supplementary_Data [file jjag011_supplementary_data.zip › Supplement_Data.zip!/Supplementary Info/UNITI Jr 40 kg Supplementary Table S1 2.23.26.docx]

**Supplementary Table S1: Definitions of select endpoints used in UNITI Jr**

| **Endpoint** | **Definition** |
| --- | --- |
| Clinical remission | PCDAI score ≤10 |
| Clinical response | Reduction from baseline in PCDAI score ≥12.5 points with a total PCDAI score not more than 30 |
| Corticosteroid-free clinical remission | PCDAI score ≤10 points and did not receive corticosteroids for at least 90 days prior to Week 52 |
| Clinical biomarker response | Reduction from baseline in PCDAI score ≥12.5 points with a total PCDAI score not more than 30 points and ≥50% reduction from baseline in CRP, fecal calprotectin or fecal lactoferrin |
| Endoscopic response | Reduction in SES-CD score ≥50% or SES-CD score ≤2, in patients with baseline  SES-CD score ≥3 |
| Endoscopic remission | SES-CD score ≤2, in patients with a baseline SES-CD score ≥3 |
| Loss of response | Increase in PCDAI >12.5 above reference PCDAI score at 2 consecutive visits ≥7 days apart from Week 8 for induction responders and Week 16 for delayed induction responders and PCDAI score is >30 at 2 consecutive visits ≥7 days  apart. |

CRP, C-reactive protein; PCDAI, Pediatric Crohn’s Disease Activity Index; SES-CD, Simple Endoscopic Score for Crohn’s Disease.
