# Supplementary material for: Ustekinumab therapy for moderately to severely active pediatric Crohn’s disease: UNITI Jr study safety and efficacy results in patients weighing at least 40 kg
Source: J Crohns Colitis. 2026 Mar 12;20(3):jjag011. doi: 10.1093/ecco-jcc/jjag011 (PMC13017028; doi:10.1093/ecco-jcc/jjag011)
Supplement: jjag011_Supplementary_Data [file jjag011_supplementary_data.zip › Supplement_Data.zip!/Supplementary Info/UNITI Jr 40 kg Supplementary Table S2 2.23.26.docx]

**Supplementary Table S2: Additional information on missing data**

| **Computation of Laboratory Score** | **Note** |
| --- | --- |
| If at the Week 8 visit the hematocrit, erythrocyte sedimentation rate or albumin values were missing but data were available for all other PCDAI components subscores (and items), then the respective laboratory test results from the previous visit at Week 0 were used to compute the laboratory score | Imputing missing Week 8 laboratory values with values from Week 0 represents a conservative approach, as it assumes no improvement in the laboratory values |

PCDAI, Pediatric Crohn’s Disease Activity Index.
