# Supplementary material for: Ustekinumab therapy for moderately to severely active pediatric Crohn’s disease: UNITI Jr study safety and efficacy results in patients weighing at least 40 kg
Source: J Crohns Colitis. 2026 Mar 12;20(3):jjag011. doi: 10.1093/ecco-jcc/jjag011 (PMC13017028; doi:10.1093/ecco-jcc/jjag011)
Supplement: jjag011_Supplementary_Data [file jjag011_supplementary_data.zip › 24-Feb-2026_071038_Supplement_Data.zip!/UNITI Jr 40 kg Supplementary Table S2.docx]

**Supplementary Table S2: Additional information on missing data**

| **Computation of Laboratory Score** | **Note** |
| --- | --- |
| If at the Week 8 visit the hematocrit, erythrocyte sedimentation rate or albumin values were missing but data were available for all other PCDAI components subscores [and items], then the respective laboratory test results from the previous visit at Week 0 were used to compute the laboratory score | Imputing missing Week 8 laboratory values with values from Week 0 represents a conservative approach, as it assumes no improvement in the laboratory values |

PCDAI, Paediatric Crohn’s Disease Activity Index.
