# Supplementary material for: Ustekinumab therapy for moderately to severely active pediatric Crohn’s disease: UNITI Jr study safety and efficacy results in patients weighing at least 40 kg
Source: J Crohns Colitis. 2026 Mar 12;20(3):jjag011. doi: 10.1093/ecco-jcc/jjag011 (PMC13017028; doi:10.1093/ecco-jcc/jjag011)
Supplement: jjag011_Supplementary_Data [file jjag011_supplementary_data.zip › 24-Feb-2026_071038_Supplement_Data.zip!/UNITI Jr 40 kg Supplementary Table S3.docx]

**Supplementary Table S3: Comparison of efficacy endpoints in paediatric and adult patients at Week 52**

| **Outcome** | **Paediatric UNITI Jr**  **[N=48]** | **Adult IM-UNITI**^13^  **[N=257]** |
| --- | --- | --- |
| **Dose** | 90 mg q8w or q12w | 90 mg q8w or q12w |
| **Clinical remission^a^, n, %  [95% CI]** | 25/48 [52.1]  [38.3, 65.5] | 131/257 [51.0] |
| **Clinical response^b^, n, %**  **[95% CI]** | 27/48 [56.3]  [42.3, 69.3] | 151/257 [58.8] |
| **Corticosteroid-free clinical remission^c^, n, % [95% CI]** | 25/48 [52.1]  [38.3, 65.5] | 111/257 [43.2] |
| **CRP, mean change from baseline, mg/L [SD]** | -11.17 [24.159] | 4.28 [20.863] |
| **CRP normalization ≤3 mg/L at Week 52^d^, n, % [95% CI]** | 8/31 [25.8]  [13.7, 43.2] | 41/193 [21.2]^e^ |
| **CRP normalization ≤5 mg/L at Week 52^d^, n [%]** | 10/29 [34.5]  [19.9, 52.7] | Not performed |
| **Fecal calprotectin, mean change from baseline, mg/kg, [SD]** | -538.20 [1271.33] | -10.85 [936.916] |
| **Fecal calprotectin <250 µg/g at Week 52^d^, n [%]** | 6/41 [14.6] | q12w: 27.5% [n=91]  q8w: 30.1% [n=83] |

^a^Clinical remission defined as a PCDAI or sPCDAI score ≤10 for paediatric study or CDAI ≤150 for adult study.

^b^Clinical response defined as reduction from baseline in PCDAI score ≥12.5 points with a total PCDAI score not more than 30 for paediatric study and reduction from baseline in CDAI score ≥100 points with a total CDAI score not more than 150 for adult study.

^c^Corticosteroid-free clinical remission defined as PCDAI score ≤10 points and not receiving corticosteroids for at least 90 days prior to Week 52 for paediatric study and CDAI <150 points in patients not receiving corticosteroids at Week 44 for adult study.

**^d^**Post-hoc analysis without intercurrent event criteria applied.

^e^Abnormal CRP defined as CRP value >3 mg/L.

CDAI, Crohn’s Disease Activity Index; CI, confidence interval; CRP, C-reactive protein; mg/kg, milligram per kilogram; mg/L, milligram per litre; PCDAI, Paediatric Crohn’s Disease Activity Index; sPCDAI, Short Paediatric Crohn’s Disease Activity Index; SD, standard deviation.
