# Supplementary material for: Ustekinumab therapy for moderately to severely active pediatric Crohn’s disease: UNITI Jr study safety and efficacy results in patients weighing at least 40 kg
Source: J Crohns Colitis. 2026 Mar 12;20(3):jjag011. doi: 10.1093/ecco-jcc/jjag011 (PMC13017028; doi:10.1093/ecco-jcc/jjag011)
Supplement: jjag011_Supplementary_Data [file jjag011_supplementary_data.zip › 24-Feb-2026_071038_Supplement_Data.zip!/UNITI Jr 40kg Supplementary Figure S1 FINAL.pdf]

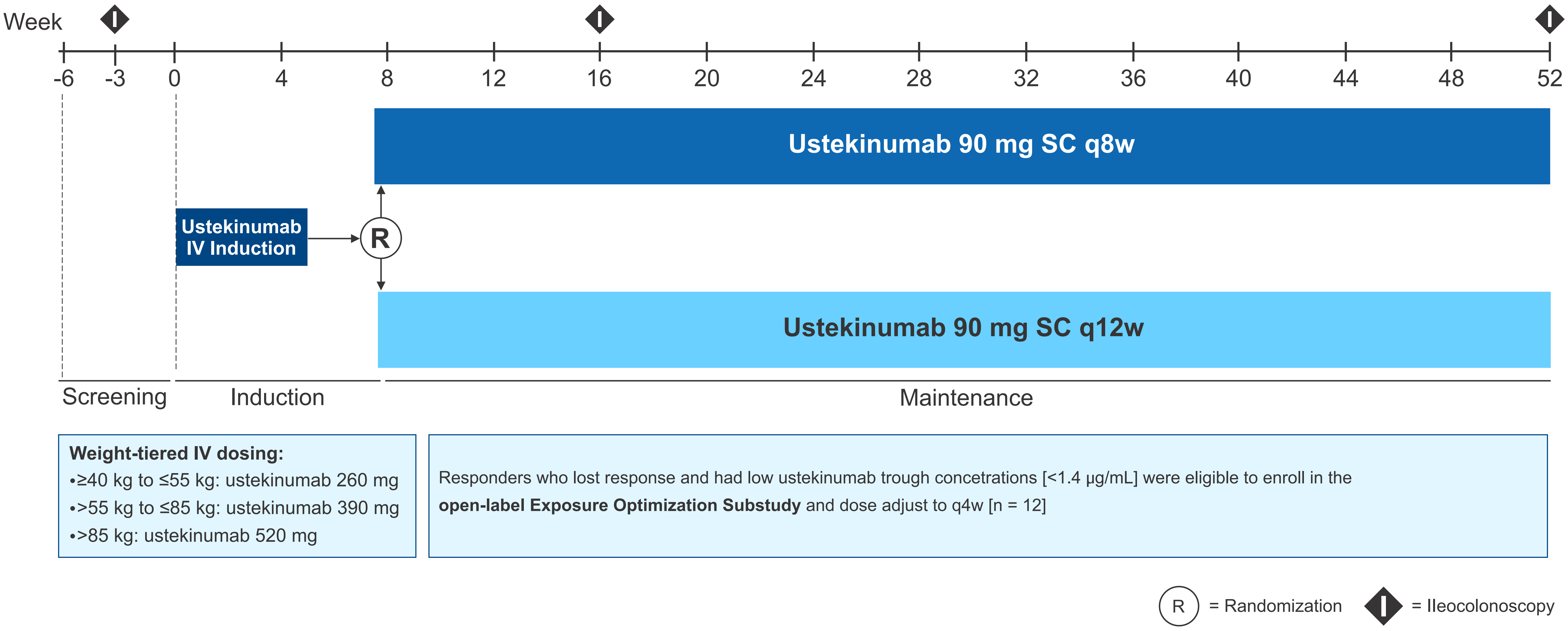
Week
-6
-3
0
4
8
12
16
20
24
28
32
36
40
44
48
52
Ustekinumab 90 mg SC q8w
Ustekinumab IV Induction
R
Ustekinumab 90 mg SC q12w
Screening
Induction
Maintenance
Weight-tiered IV dosing:
•≥40 kg to ≤55 kg: ustekinumab 260 mg
•>55 kg to ≤85 kg: ustekinumab 390 mg
•>85 kg: ustekinumab 520 mg
Responders who lost response and had low ustekinumab trough concetrations [<1.4 µg/mL] were eligible to enroll in the open-label Exposure Optimization Substudy and dose adjust to q4w [n = 12]
R = Randomization
= Ileocolonoscopy
